# Immediate Kangaroo Mother Care and Survival of Low Birth Weight Infants

**DOI:** 10.1056/NEJMoa2026486

**Published:** 2021-05-27

**Authors:** WHO Immediate KMC Study Group

## Abstract

**Background:**

Kangaroo Mother Care initiated after stabilization reduces mortality in infants with birthweight <2.0 kg, but the majority of deaths occur before stabilization. The safety and efficacy of Kangaroo Mother Care initiated soon after birth is uncertain.

**Methods:**

We conducted a randomized controlled trial in five hospitals in Ghana, India, Malawi, Nigeria, and Tanzania. Infants with birth weight between 1.0 and <1.8 kg were randomly assigned to immediate Kangaroo Mother Care (intervention) or to conventional care until stabilization, and Kangaroo Mother Care thereafter (control). The primary outcomes were deaths in the neonatal period (first 28 days of life) and in the first 72 hours of life. The study was stopped early on the recommendation of the DSMB owing to reduced neonatal mortality with the intervention.

**Results:**

A total of 3211 infants and their mothers were randomly allocated (1609 intervention, 1602 control group). The median daily duration of skin-to-skin contact in neonatal intensive care units was 16.9 hours (IQR 13.0–19.7) in the intervention and 1.5 hours (IQR 0.3–3.3) in control group. Neonatal death occurred in 191 infants (12.0%) and 249 (15.7%) infants, respectively (RR 0.75; 95% CI 0.64–0.89; p=0.001);death in the first 72 hours of life occurred in 74 infants (4.6%) and 92 infants (5.8%), respectively (RR 0.77, 95% CI 0.58–1.04; p=0.09).

**Conclusion:**

In infants with birthweight between 1.0 and <1.8 kg, immediate Kangaroo Mother Care (versus conventional care) resulted in a significant reduction in neonatal mortality, but not in mortality within the first 72 hours.

## INTRODUCTION

Low birth weight (LBW) infants, born preterm and/or small for gestational age, constitute about 15% of neonates, but account for 70% of all neonatal deaths. Reducing deaths in LBW infants, particularly in low- and middle- income countries (LMICs) in Asia and Sub-Saharan Africa, is therefore key to the achievement of the Sustainable Development Goal target of reducing neonatal mortality to <12/1000 live births in each country by 2030.^1–3^

Kangaroo Mother Care, defined as continuous skin-to-skin contact of the baby with the mother’s chest and exclusive breastmilk feeding, is one of the most effective interventions for preventing mortality of LBW infants.^4^ World Health Organization (WHO) ^5^ guidelines currently recommend initiation of short intermittent Kangaroo Mother Care sessions when the infant’s condition begins to stabilize, and continuous Kangaroo Mother Care when fully stable. A Cochrane review reported a 40% reduction in mortality in LBW infants given Kangaroo Mother Care after stabilization compared to conventional care in hospitals (3.2% versus 5.3%; risk ratio (RR) 0.60, 95% confidence interval (CI) 0.39 to 0.92; eight trials, 1736 infants).^6^ This review also showed fewer infections, higher exclusive breastfeeding and better weight gain in infants who received Kangaroo Mother Care. In studies included in the review, the mean age at randomization (when infants were considered stable) ranged from 10 hours to 24.5 days of life. About 45% of neonatal deaths occur within 24 hours of birth and 80% within the first week of life; ^7^ thus the majority of deaths among LBW infants occur before Kangaroo Mother Care can be initiated.

Two randomized controlled trials (RCTs) have evaluated the effect of initiating Kangaroo Mother Care immediately after birth on physiological stabilization. In South Africa ^8^ and Vietnam^9^, skin-to-skin contact started soon after birth in LBW infants resulted in earlier stabilization than conventional care.

There is a critical knowledge gap regarding the effect of initiating continuous Kangaroo Mother Care soon after birth before stabilization on mortality in LBW infants. We conducted this large RCT to evaluate the safety and efficacy of continuous Kangaroo Mother Care initiated immediately after birth in infants with a birthweight of 1.0 to <1.8 kg.

## METHODS

The details of the study methods have been published previously and are briefly summarized here.^10^

### Study design and participants

This multi-center, non-blinded RCT was undertaken in five tertiary-level hospitals in Ghana, India, Malawi, Nigeria and Tanzania. All liveborn infants in the participating hospitals, with birthweight between 1.0 to <1.8kg regardless of their gestational age, mode of delivery or singleton/twin status, were eligible for inclusion. Mother-infant(s) pairs were excluded if the mother was <15 years old, unable or unwilling to provide consent, had triplets or more, was sick and unlikely to be able to provide Kangaroo Mother Care within the first 3 days after birth, could not be enrolled within 2 hours of childbirth or resided outside the study area. Infants who were unable to breathe spontaneously by one hour of age or had a major congenital malformation were also excluded.

This trial was approved by Ethics Review Committees at WHO and at each site. The study was overseen by a steering committee and a Data and Safety Monitoring Board (DSMB). RB, SR, SY and NM vouch for the accuracy and completeness of the data and for the fidelity of the trial to the protocol.

### Study procedures

Three independent teams, trained in study standard operating procedures, were responsible for (1) screening and enrolment (2) Kangaroo Mother Care support and (3) outcome measurement at each site.

Pre-screening of all pregnant women admitted for childbirth was conducted to identify women at high risk of delivering a LBW infant and consent for study participation was sought. All infants born in the hospital were weighed and screened for eligibility. If the mother and infant were eligible, consent was confirmed if it had already been obtained before birth. If consent could not be obtained before birth, it was obtained after birth. At enrolment, mothers were asked to identify one or two adult women who could act as their surrogates for providing Kangaroo Mother Care; only women are permitted to stay in postnatal areas in all study hospitals.

Randomization was performed using a computer-generated block list, with variable block size, stratified by site and birthweight 1.0 to <1.5kg and 1.5 to <1.8kg. The random allocation was sealed in serially numbered, opaque envelopes prepared at the WHO and delivered to the sites. A research assistant conducted randomization by opening the next numbered envelope. Twins were allocated to the same group. The nature of the intervention prevented blinding, but outcome assessment was done by an independent team not involved in intervention delivery.

System changes in obstetric and neonatal care as well as structural changes to the neonatal intensive care unit (NICU) were necessary. Mother-NICU, which included mothers’ beds and reclining chairs, were built or converted from an existing NICU. All equipment, staff and care provision in the Mother-NICU remained the same as the NICU. The infant was secured firmly to the mother’s chest with a binder that ensured a patent airway.^11^ All care to the mother and infant were provided while in skin-to-skin contact if possible, all interruptions were documented. Obstetricians supervised essential postpartum care provided to mothers in the Mother-NICU.

Infants allocated to control group were transferred to the NICU without the mother, following the standard care practices. The mother provided expressed breast milk, and brief sessions of Kangaroo Mother Care when the infant started to recover and was at least 24 hours old.

Hospital staff provided care for all enrolled infants according to the WHO minimum care package for small infants.^12^ In both intervention and control groups, once clinically stable based on pre-specified criteria^10^ for 24 hours, the infant was shifted from Mother-NICU or NICU to Kangaroo Mother Care ward where continuous Kangaroo Mother Care was provided until discharge.

### Outcomes and their measurement

The primary outcomes were mortality from enrolment to 28 days of age and mortality from enrolment to 72 hours of age. Secondary outcomes included hypothermia (any axillary temperature < 36°C), hypoglycemia (any blood glucose level < 45 mg/dl, measured when clinically indicated), suspected sepsis, time to clinical stabilization, fully breastfed (only by suckling) at the time of discharge, exclusive breastfeeding at the end of neonatal period, maternal satisfaction with care and maternal depression (supplementary Table S1).^10^ Additionally, mortality from birth to 72 hours in non-enrolled infants 1.0 to <1.8 kg was documented. The only serious adverse event assessed according to the protocol was death. Outcome data were collected using identical methods and procedures for all enrolled infants. Clinical monitoring was done every 6 hours for all infants while they were in Mother-NICU or NICU. Information on duration of skin-to-skin contact and duration of hospital stay was collected by research assistants. A home visit was performed on day 29 for data on survival, breastfeeding and maternal depression.

### Statistical analysis

We estimated that 4200 infants were needed to detect a 20% relative mortality reduction at 28 days (16.8% mortality in intervention group compared with 21.1% in control group), with 95% confidence level and 90% power and 10% loss to follow-up. The DSMB conducted interim analyses at 50% and 75% enrolment. After the second interim analysis, the DSMB recommended stopping enrolment in the trial because of a clear benefit in neonatal survival. (See supplementary appendix.)

Intention-to-treat analyses were performed for primary and secondary outcomes.^10^ Risk ratios and 95% CI were calculated for the outcomes. Adjusted risk ratios were estimated using log-binomial regression controlled for clustering due to multiple births and other important baseline characteristics which could be potential confounders. Hazard ratios were calculated using multivariable Cox survival analysis to compare time to clinical stabilization between groups. We used marginal mean imputation for missing values in continuous covariates and the most frequent response to impute categorical variables. No imputation was made for the primary outcomes.

Pre-specified subgroup analyses were performed to explore modification of effect of immediate Kangaroo Mother Care on primary outcomes by birthweight (1.0 to <1.2kg, 1.2 to <1.5 kg, 1.5 to <1.8 kg), gestational age (<31, 31 to <34, 34 to <37, ≥37 weeks), mode of delivery (vaginal birth, Caesarean section), singleton/twin gestation, and size for gestational age (small for gestational age, not small for gestational age).^10^ Subgroup analyses by site was conducted *post-hoc*. In the intervention group, we examined the primary outcomes in subgroups by compliance to Kangaroo Mother Care (skin-to-skin contact for ≥20 hours, 10–19 hours, and <10 hours per day). To address reverse causality in this analysis, we excluded infants with any sign of severe illness in the first 6 hours of life. Causes of death were assigned by investigators based on clinical information for hospital deaths and by verbal autopsy for deaths at home after discharge.

*Post hoc* analyses were conducted to explore the effect of the intervention on breastfeeding during hospital stay, including proportion of newborns by group who had initiated breastmilk feeds within 24 hours, were put to breast in the first 72 hours, reached full breastmilk feeds within 7 days, and were discharged on exclusive breastmilk feeding.

## RESULTS

**Figure 1.**
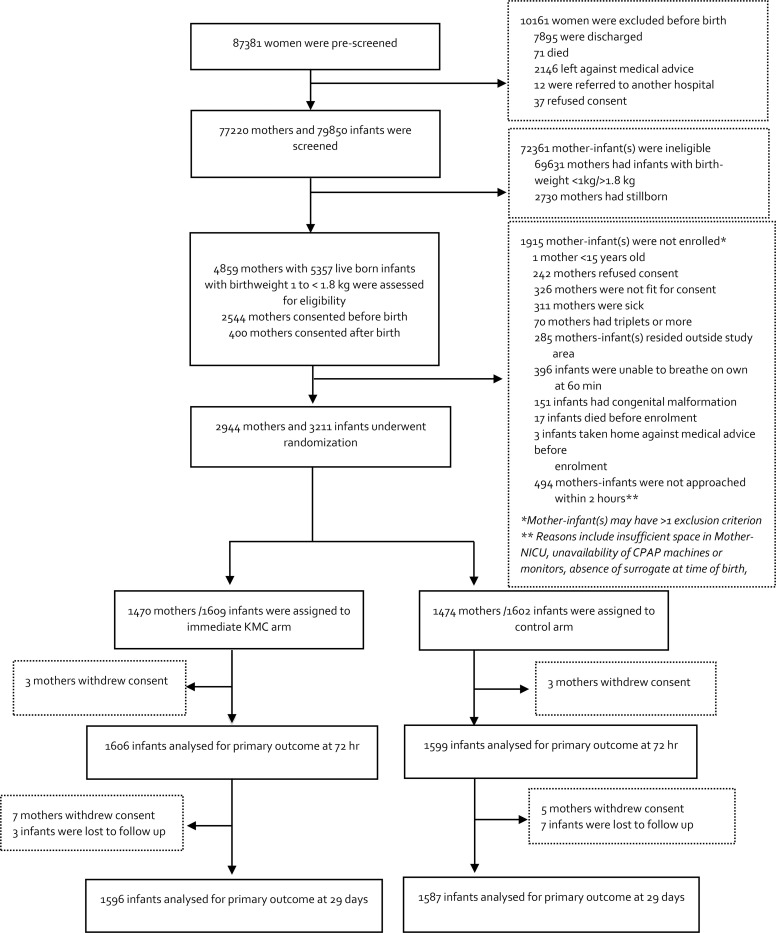
Participants flowchart

A total of 87,381 pregnant women were pre-screened and 79,850 infants were screened for eligibility between 30 November 2017 and 20 January 2020, of which 5357 infants (from 4859 mothers) met the weight criteria for enrolment. Of them, 3211 infants (2944 mothers) were randomly allocated - 1609 infants (1470 mothers) to the intervention group and 1602 infants (1474 mothers) to the control group (Figure 1).

Table 1 and Table S2 show the baseline characteristics of randomized infants, their mothers and their families. Socio-demographic, newborn and maternal characteristics were similar in both groups. The mean gestation was 32.6 weeks and mean birthweight was 1.5 kg in both groups. Only 0.3% randomized infants had missing observations for covariates, except family income which was missing for 8%.

The median age of initiation of skin-to-skin contact was 1.3 hours (IQR 0.8–2.7) in intervention group, and 53.6 hours (IQR 33.8 – 101.4) in control group. The duration of NICU stay was similar in intervention and control groups (median 6.4 days in both groups). During NICU stay, median daily duration of skin-to-skin contact was 16.9 hours (and 1.5 hours, respectively. The daily duration on each day in the first two weeks is given in Table S3. The main reasons for not being in skin-to-skin contact in the intervention group were medical procedures, infant care and routine activities of mother. The median daily duration of skin-to-skin contact in the Kangaroo Mother Care ward was similar in both groups (20.2 hours vs 19.0 hours) (Table 2).

From enrolment to 28 days of age, 191 infants (12.0%) in the intervention group and 249 (15.7%) in the control group died (RR 0.75, 95% CI 0.64–0.89; p=0.001). The number needed to treat was 27 (95% CI 17–77) to prevent one death. From enrolment to 72 hours of age, 74 infants (4.6%) in intervention and 92 infants (5.8%) in control group died (RR 0.77, 95% CI 0.58–1.04; p=0.09) (Table 3).

**Figure 2.**
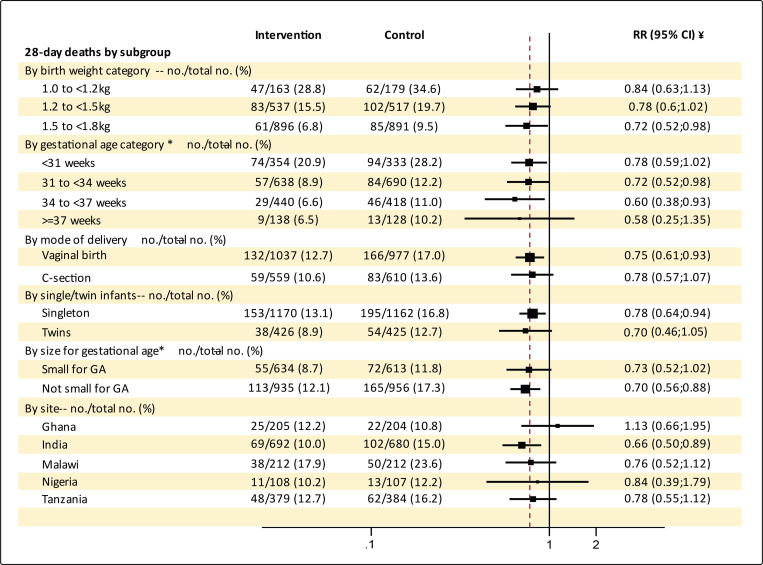
Subgroup analyses of primary outcomes by birthweight, gestational age, multiple pregnancy, mode of delivery and size for gestational age * 26 infants in the intervention and 18 infants in the control group have their gestational age at birth missing. Size for gestational age could not be calculated for one additional infant that was born with indeterminate sex. ¥ adjusted by site and clustering due to multiple births. For the subgroup analysis by site, adjustment was only done for clustering due to multiple births. **The widths of the confidence intervals were not adjusted for multiplicity, so the intervals should not be used to infer definitive intervention effects. The size of squares representing the point estimates is proportional to the weight assigned to the subgroup.

The intervention had similar effects across birthweights, gestation and weight for gestational age categories, different modes of delivery, and singletons or twins. (Figure 2 and Figure S1). All sites showed benefit in their point estimates except Ghana. In the intervention group, the risk of death was lower in infants who received more hours of skin-to-skin contact per day (Table S4). Most deaths were caused by sepsis and preterm birth complications. Sepsis-associated mortality was 4.4% in the intervention group and 6.9% in the control group (RR 0.64, 95% CI 0.48 to 0.86). (Table S5).

Secondary outcome results are presented in Table 3. The proportion of infants with suspected sepsis was 22.9% in intervention group and 27.8% in control group (adjusted RR 0.82, 95% CI 0.73–0.93); hypothermia was documented in 5.6% and 8.3%, respectively (adjusted RR 0.65, 95%CI 0.51–0.83). The time to stabilization and incidences of hypoglycemia, feeding fully by suckling at the time of discharge, and exclusive breastfeeding at the end of neonatal period were similar in both groups. In *post-hoc* analyses, breastmilk feeding was initiated within the first 24 hours in 58.5% vs 45.5% of infants, and full breastmilk feeding within 7 days was achieved in 78.4% vs 69.0%, respectively (Table S6). Of 2146 infants with birthweight between 1.0 and <1.8 kg who were not enrolled in the trial, 340 (15.8%) died by 72 hours.

## DISCUSSION

This multicenter trial demonstrated that initiation of continuous Kangaroo Mother Care soon after birth for infants with birthweight between 1.0 to <1.8 kg improved neonatal survival by 25%, compared with Kangaroo Mother Care initiated after stabilization, as is currently recommended. The intervention would need to be provided to 27 infants (95% CI 17–77) to prevent one neonatal death. Implementation of the intervention required the mother, or a surrogate, to be with the baby all the time which required establishment of Mother-NICUs. The lower observed rates of hypothermia and suspected sepsis, though not adjusted for multiplicity, are consistent with results for the primary outcome and may at least in part explain the mortality benefits of immediate Kangaroo Mother Care.

Findings for the primary outcome and for infection and hypothermia were similar to those reported in earlier trials of the use of Kangaroo Mother Care in clinically stable infants.^6^ However, we did not find differences between the intervention and control groups in the two pre-specified feeding outcomes—being fully breastfed by suckling at discharge and exclusive breastfeeding at the end of the neonatal period, despite post-hoc analyses suggesting higher rates of initiation of breast-milk feeding within 24 hours, putting the baby to the breast within 72 hours and reaching full breast-milk feeding within 7 days of birth in the intervention group. We also did not find a material difference between groups in the time to stabilization, unlike two previous RCTs of a similar intervention. ^8,9^ As compared to the studies that achieved intermittent Kangaroo Mother Care in the Cochrane review ^6^, we achieved high compliance with the intervention, i.e., about 17 hours of skin-to-skin contact per day.

There are several possible mechanisms by which immediate Kangaroo Mother Care might confer benefit. As the mother and baby are in close contact from birth, the baby is more likely to be colonized by the mother’s protective microbiome, more likely to receive early breastmilk feeding and there is less handling of the baby, thus reducing the risk of infection.^13–19^ Constant monitoring of the infant by the mother, more frequent glucose monitoring, and decreased stress^20^ of mother-infant separation could also contribute to reduced mortality. Further studies in well-resourced settings could help to determine to what extent these enhanced survival results from LMIC settings are relevant to low-mortality settings with intensive infant monitoring. We observed that the risk of death was lower in infants who received more hours of skin-to-skin contact per day. However, this association is subject to confounding by medical issues in the infant that may have precluded prolonged skin-to-skin contact.

The results of this study are generalizable to most hospitals in low-resource settings where immediate-Kangaroo Mother Care can be implemented as described here. Challenges in scaling up of this intervention would include multiple stakeholders’ involvement, establishment of Mother-NICUs, a strong collaboration between obstetric and neonatal departments, and policy changes allowing surrogates to provide Kangaroo Mother Care.

Some limitations merit discussion. The nature of the intervention made blinding impossible. However, ensuring allocation concealment until completion of enrolment, rigorous adherence to predefined protocol and choice of mortality as a primary outcome minimize measurement bias. The open label design may have resulted in measurement bias in some of the secondary outcomes, which were more subjective, but would not affect our primary mortality outcomes. There was heterogeneity in the infrastructure, staff, practices and possible differences in patient profile across sites; however, this should increase the generalizability of our findings. It is not possible to partition the beneficial effect of the intervention between immediate initiation of Kangaroo Mother Care and simply the presence of the mother with her baby, because both are integral part of the intervention. Finally, approximately 20% of infants 1.0 to <1.8 kg born in study hospitals were not enrolled because mother or newborn was too sick for this intervention, which needs to be considered in estimating the potential public health impact of the intervention.

In summary, in this large, multi-site, multi-country study, conducted in low resource hospitals, continuous Kangaroo Mother Care initiated immediately after birth in infants with a birthweight of 1.0 to <1.8 kg resulted in a significantly lower risk of neonatal death, although not of death within 72 hours, compared to the current WHO recommendation of initiating Kangaroo Mother Care after stabilization.

## Supplementary Material

Supplement

## Figures and Tables

**Table 1.** Baseline characteristics of randomized infants, mothers and households

	Immediate Kangaroo Mother Care	Control
**Infant’s characteristics** #	**N=1609**	**N=1602**
Age at randomization in minutes (median, IQR)	35 (20,55)	33 (20,54)
Birth weight in kg, mean (SD)	1.5 (0.2)	1.5 (0.2)
Gestational age at birth, mean (SD)*¥	32.6 (3.0)	32.6 (2.8)
Male, n (%)	752 (46.7)	748 (46.7)
Infants born as twin, n (%)	430 (26.7)	430 (26.8)
Delivery by C-section n (%)	559 (34.7)	614 (38.3)
Site, n (%)		
Ghana	205 (12.7)	205 (12.8)
India	695 (43.2)	682 (42.6)
Malawi	217 (13.5)	222 (13.9)
Nigeria	108 (6.7)	107 (6.7)
Tanzania	384 (23.9)	386 (24.1)
**Mother and household’s characteristics** #	**N=1470**	**N=1474**
Mother’s age in years, mean (SD)	26.7 (5.8)	26.7 (5.8)
Mother's years of schooling, median (IQR) §	10 (7,12)	10 (7,12)
Family income in US dollars, median (IQR)	168 (110,285)	176 (110,280)
Piped water as main source of drinking water,n (%)	934 (63.5)	953 (64.7)
Households with a toilet in the house, n (%) §	1288 (87.9)	1343 (91.3)

# There were 534 infants (from 267 mothers) who were born from a multiple pregnancy and both were eligible and enrolled (278 infants in the intervention and 256 infants in the control).

In addition, there were 325 mothers with multiple pregnancies in whom only one of the infants was eligible and the other one was ineligible (152 infants in the intervention group and 173 in the control).

* Gestational age based on ultrasound in first or second trimester, and if not available then based on LMP, and if both USG and LMP not available, then based on Ballard score (assessing measures of maturity on examination)^21^

¥ Gestational age at birth missing for 27 infants in intervention and 18 infants in control group

§ 2 households in intervention and 2 in control group have missing data on mother’s education

5 households in intervention and 3 in control group have missing data on availability of toilet Additional baseline characteristics are provided in Table S1

**Table 2.** Initiation and duration of skin -to-skin contact in randomized infants

	Immediate Kangaroo Mother Care N=1609	Control N=1602
Time to initiation of skin-to-skin contact in hours* median (IQR)	1.3 (0.8–2.7)	53.6 (33.8–101.4)
Time to initiation of skin-to-skin contact by category, n (%)		
<2 hours	1098 (68.2%)	4 (0.2%)
2 to ≤6 hours	306 (19.0%)	14 (0.9%)
6 to ≤12 hours	94 (5.8%)	14 (0.9%)
12 to ≤24 hours	62 (3.9%)	74 (4.6%)
24 to ≤168 hours	32 (2.0%)	1176 (73.4%)
>168 hours to end of neonatal period	1 (0.1%)	142 (8.9%)
Never initiated	16 (1.0%)	178 (11.1%)
Skin-to-skin contact while in NICU, hours per day, median (IQR)	1609	1602
Overall	16.9 (13.0–19.7)	1.5 (0.3–3.3)
With mother	12.3 (6.8–16.5)	1.5 (0.2–3.2)
With surrogate	2.3 (0.1–6.5)	0 (0–0)
Skin-to-skin contact while in Kangaroo Mother Care ward, hours per day, median (IQR)	1300	1224
Overall	20.2 (18.6–21.3)	19.0 (16.3–20.4)
With mother	19.4 (14.8–20.6)	18.0 (14.1–19.9)
With surrogate	0 (0–0.85)	0 (0–0)

* If the infant never initiated skin-to-skin contact and: (i) died: censored at the time of death (ii) taken home against medical advice or refused consent: censored at time of leaving the hospital or refusing consent, respectively; (iii) was discharged: censored at time of discharge; (iv) was still in hospital at the end of the neonatal period: censored at day 28.

**Table 3.** Primary and secondary outcomes in randomized infants

	Immediate Kangaroo Mother Care (1609 assigned)	Control (1602 assigned)	Adjusted RR (95%CI)*	P
**Primary outcomes**				
Death between enrolment and 28 days of age, n (%)	191/1596 (12.0%)	249/1587 (15.7%)	0.75 (0.64–0.89)	0.001
Death between enrolment and 72hr of age, n (%)	74/1606 (4.6%)	92/1599 (5.8%)	0.77 (0.58–1.04)	0.09
**Secondary outcomes** †				
Exclusive breastfeeding at the end of neonatal period, n (%)	1208/1401 (86.2%)	1140/1336 (85.3%)	1.01 (0.98–1.05)	
Fully breastfed (only by suckling) at hospital discharge, n (%)	62/1435 (4.3%)	55/1376 (4.0%)	1.06 (0.73–1.53)	
Hypothermia, n (%)^1^	90/1609 (5.6%)	133/1602 (8.3%)	0.65 (0.51–0.83)	
Time to clinical stabilization in hr, median (IQR) ^2^	73.8 (26.8;138.5) (n=1609)	74.8 (25.3;140.6) (n=1602)	0.98 (0.90; 1.07) §	
Suspected sepsis, n (%) ^3^	361/1575 (22.9%)	434/1561 (27.8%)	0.82 (0.73–0.93)	
Hypoglycemia at any time between 0–36h of age, n (%)^4^	82/799 (10.3%)	66/651 (10.1%)	1.15 (0.85–1.56)	
Duration of hospital stay in days, mean (SD)^5^	14.9 (0.2) (n=1609)	15.2 (0.2) (n=1602)	1.07 (0.99;1.16) §	
Maternal satisfaction with health care in the hospital, mean (SD)^6^	9.2 (1.0) (n=1282)	9.1 (1.2) (n=1233)	0.11 (0.03–0.19) ¥	
Maternal depression, n (%)^7^	2/1276 (0.2%)	7/1231 (0.6%)	0.23 (0.05–1.14)	

1 Any instance of axillary temperature <36ºC at any time from 2 hours after randomization until discharge from hospital.

2 First time at which the infant had all signs of clinical stability: no need for CPAP, no episodes of apnea, SpO_2_>90, Respiratory rate 40 to < 60, Heart rate 80 to < 180 bpm, Temperature between 36 to 37.4ºC, and no need for IV fluids.

3 Suspected sepsis defined as one or more of the following signs/symptoms: temperature<35.5ºC or > 38ºC, no movement or movement only on stimulation, chest indrawing, convulsions. For all the signs/symptoms, we removed the first 24 hours, after that time the child should have been well for at least 24 hours before becoming sick. Denominator excludes infants that died, LAMA or were discharged before 48 hours of age.

4 Hypoglycemia defined as blood sugar < 45 m/dl or < 2.6 mmol/L measured when clinically indicated

5 Duration of hospital stay was a pre-specified process outcome

6 Maternal satisfaction with health care in the hospital was collected at discharge on a score of 1 to 10. Higher score implies higher satisfaction

7 Maternal depression defined as a score of >15 points on Patient Health Questionnaire 9

* adjusted for clustering due to multiple births, site, delivery mode, multiple pregnancy, age at randomization, infant’s sex, infant’s weight, mother's years of schooling, maternal age, households with toilet in the house, and family income.

† The 95% confidence intervals for secondary outcomes are not adjusted for multiplicity and should not be used to infer definitive intervention effects.

§ Hazard ratio

¥ Mean difference
