# Supplementary material for: Immediate Kangaroo Mother Care and Survival of Low Birth Weight Infants
Source: N Engl J Med. Author manuscript; Available in PMC 2021 May 27. (PMC8108485; doi:10.1056/NEJMoa2026486)
Supplement: Supplement [file NEJMoa2026486-supplement.pdf]

Supplementary Appendix

Supplement to: WHO Immediate KMC Study Group. Immediate Kangaroo Mother Care and Survival of Low Birth Weight Infants

Table of contents

Supplementary Appendix 1

WHO Immediate Kangaroo Mother Care Study Group 3

Statistical analysis plan 5

Sample size 5

Statistical analysis 5

Data and safety monitoring board (DSMB) 8

Supplementary tables and figures 10

Figure S1. Subgroup analyses of 72hr mortality by birth weight, gestational age, multiple pregnancy, mode of delivery, size for gestational age and site 10

Table S1. Primary and secondary outcomes 11

Table S2. Additional baseline characteristics of randomized infants, mothers and households 13

Table S3. Duration of skin-to-skin contact in hours per day while the newborns were in the NICU during the first 2 weeks of life 15

Table S4. Neonatal mortality in Intervention group by hours of skin-to-skin contact per day 16

Table S5. Cause-specific neonatal mortality in randomized infants 17

Table S6. Additional breastfeeding outcomes (post-hoc analyses) 18

Table S7. Risk factors for low birth weight in the pre-Screening form 19

WHO Immediate Kangaroo Mother Care Study Group

Authors listed by site:

Ghana- Sam Newton[1], Roderick Larsen-Reindorf[1], Adwoa Boakye-Yiadom[2], Naana Wireko-Brobby[2], Isaac Nyanor[2], Alexander Ansah Manu[3], Daniel Ansong[1], Gyikua Plange-Rhule[1].

India- Sugandha Arya[4], Pratima Mittal[4], Nidhi Chopra[4], Jyotsna Suri[4], M Jeeva Sankar[5], Pratima Anand[4], Kashika Nagpal [4], Isha Saini[4], K C Aggarwal[4], Nitya Wadhwa[6], Harish Chellani[4].

Malawi- Kondwani Kawaza[7,8], Luis Gadama[7,8], Ausbert Thoko Msusa[7,8], Vincent Samuel[7], Queen Dube[7,8]

Nigeria- Chineme Henry Anyabolu[9], Oluwafemi Kuti[9], Bankole Peter Kuti[9], Ebunoluwa Aderonke Adejuyigbe[9]

Tanzania- Helga Naburi[10], Evelyne Assenga[10], Matilda Ngarina[11], Robert Moshiro[11], Augustine Massawe[10]

Intervention support and monitoring team: Nils Bergman[12], Agnes Linnér[12], Barak Morgan[13], Siren Rettedal[14], Björn Westrup[12]

WHO trial coordination team: Suman Rao[15], Sachiyo Yoshida[15], Harsh Jaiswal[15], Hiresh Tiwary[15], Nicole Minckas[15], Rajiv Bahl[15]

Affiliations:

1. Kwame Nkrumah University of Science and Technology, Kumasi, Ghana
2. Komfo Anokye Teaching Hospital, Kumasi, Ghana
3. School of Public Health, University Of Ghana
4. Vardhaman Mahavir Medical College and Safdarjung Hospital, New Delhi, India
5. All India Institute of Medical Sciences, New Delhi, India
6. Translational Health Science and Technology Institute, Faridabad, India
7. University Of Malawi, College Of Medicine, Blantyre, Malawi
8. Queen Elizabeth Central Hospital, Blantyre, Malawi

9. Obafemi Awolowo University, Ile-Ife, Nigeria
10. Muhimbili University of Health and Allied Sciences, Dar es Salaam, Tanzania
11. Muhimbili National Hospital, Dar es Salaam, Tanzania
12. Karolinska Institute, Stockholm, Sweden
13. Institute for Safety Governance and Criminology, University of Cape Town. South Africa
14. Stavanger University, Stavanger, Norway.
15. Department of Maternal, Newborn, Child and Adolescent Health and Ageing, World Health Organization, Geneva, Switzerland

Statistical analysis plan

Sample size

We estimated the sample size on the basis of the primary outcome neonatal mortality at 28 completed days with a two-sided 5% significance level test and a power of 90%. A total of about 4,200 babies, accounting for a 10% loss to follow-up, were needed to detect a 20% relative mortality reduction or more from 21.1% to 16.8% among infants with birth weights from 1.0 to less than 1.8 kg receiving immediate Kangaroo Mother Care.

Statistical analysis

The primary analysis was performed according to the intention-to-treat principle based on all participants with outcome data available. Even if Kangaroo Mother Care was discontinued because of clinical conditions of the mother or infant, the infant was not excluded. Effect size was estimated with comparison of intervention and control arms mortality risks. The two primary outcomes are complementary, so adjustment for type I error was not needed. No imputation of primary outcomes was needed as mortality information was available for all enrolled infants.

Baseline characteristics were compared between groups to detect imbalances in prognostic variables that could bias the results. These data include maternal age, parents' schooling, family income, household characteristics, mode of delivery, birth weight, Apgar score, and age at randomization. We summarized continuous variables as means and standard deviations and categorical variables as frequencies and percentages. We used marginal mean imputation for missing values in covariates. Means were used to impute continuous variables and the most frequent response was used to impute categorical variables.

The intervention arm was compared against the control arm for the primary and secondary outcomes using risk ratios with 95% confidence intervals. For binary outcomes, the statistical technique used to

conduct tests and obtain confidence intervals was a logistic model with a binomial distribution and the log link to obtain relative risks. The variable study site, a design variable, was included in the model, as well as a clustering feature for multiple births. For continuous outcomes, the intervention arm was compared against the control arm using hazard ratios and 95% confidence intervals or mean differences and 95% confidence intervals. The statistical technique used to conduct tests and obtain confidence intervals for this type of variables was a multivariable Cox proportional hazards models or general linear model including study facility in the model as stratifying variable, as well as a clustering feature for multiple births. Separate models were fitted for each of the primary and secondary outcomes.

All models were fitted using STATA/MP Software version 14.0

The effect of Kangaroo Mother Care on the probability of neonatal death was assessed using a logistic model.

Variables:

- Response: neonatal death before 72 hours and neonatal death before 28 days of age

- Intervention (randomized): immediate Kangaroo Mother Care and conventional Kangaroo Mother Care

- Site: (randomization was done within sites)

- Covariates: delivery mode, multiple pregnancy, age at randomization, baby's sex, baby's weight, mother's years of schooling, maternal age, type of toilet, and family income

We conducted the following planned stratified analyses for the two mortality outcomes:

1) Birth weight categories: 1.0 to <1.2, 1.2 to <1.5 and 1.5 to <1.8 kg,

2) Gestational age at birth: less than 34 weeks vs. 34 to less than 37 weeks vs. >=37 weeks

3) Number of babies: singleton vs twin births

4) Small for gestational age: yes vs. no

5) Mode of delivery: vaginal birth vs cesarean section

Statistical tests of interaction were used to interpret whether the effect sizes in the categories were different or similar.

A secondary analysis stratified by compliance to immediate Kangaroo Mother Care over 72 hours of age was carried out. This analysis reports on efficacy of the intervention by average duration of skin-to-skin contact, classified as more than 20 hours per day, 10–19 hours per day, and less than 10 hours per day. In this secondary analysis, reverse causality was an important issue because severely ill infants could receive less or no skin-to-skin contact. To reduce the possibility of reverse causality, in a sub-analysis we excluded the infants who showed signs of severe illness in the first 6 hours of life.

Finally, an exercise was undertaken to determine the final single cause of neonatal death in the trial. Final causes of death were assigned by investigators based on clinical information for hospital deaths and by verbal autopsy for deaths at home after discharge from hospital. The WHO Newborn Health team reviewed all 440 neonatal deaths based on the forms completed at each site. Each neonatal death was assigned one underlying cause of death based on the following processes: neonatal sepsis, preterm birth complications (including Respiratory Distress Syndrome, intraventricular haemorrhage or necrotizing enterocolitis), perinatal asphyxia, congenital malformations, sudden death, other specific cause or undetermined. The site-specific list of cause of death was reviewed by the neonatal Principal Investigators at the respective sites and compared with the source documents. The changes suggested by the PIs were made. The list of final cause of death was shared with the statistical analysis team to determine cause specific mortality by study arms.

Data and safety monitoring board (DSMB)

An independent DSMB was established by the WHO including seven members with expertise in clinical trials, statistics, newborn care, and ethics in resource-limited settings. The DSMB served as the technical advisory group throughout the trial. The DSMB was responsible for safeguarding the interests of trial participants, potential participants, investigators, and sponsors; assessing the safety and early efficacy of the trial's intervention according to data available at a predefined schedule; monitoring the trial's overall conduct and quality and protecting its validity and credibility; and making recommendations concerning continuation/termination of study determined. For early efficacy, a p-value of 0.001 was planned for recommending early stopping, according to the Haybittle-Peto rule; this was applied to each of the primary outcomes, (ie There was no plan to split the alpha for the 2 primary outcomes.) . On the other hand, a p-value of 0.05 would have been considered sufficient to stop the trial in case of harm.

The DSMB conducted two interim analyses, at 50% and 75% enrolment. The DSMB conducted the second interim analysis on data of 3033 infants enrolled up to 9 December (1519 in intervention and 1514 in control). There were 173 (11.4%) neonatal deaths in the intervention group and 228 (15.1%) neonatal deaths in the control group (Adjusted RR 0.76, 95% CI 0.63-0.91, p=0.003). Before a final decision on stopping or continuing the trial, the DSMB requested to look at data of all infants enrolled up to 10 January 2020. Until this date, 3124 infants had been enrolled (1566 in intervention and 1558 in control groups) and there were 183 (11.7%) neonatal deaths in the intervention and 240 (15.4%) neonatal deaths in the control group (Adjusted RR 0.75, 95% CI 0.62-0.89, p=0.001).

On 16 January 2020, the DSMB recommended to stop the trial on the basis of clear and strong evidence of a benefit of immediate Kangaroo Mother Care and a concern that continuing to enroll given these results would be unethical.

Recruitment was stopped in all sites on the 20th of January 2020 and data collection was completed at the end of February 2020. All ethics committees and regulatory authorities were informed of the decision to stop the trial.

Supplementary tables and figures

Figure S1. Subgroup analyses of 72hr mortality by birth weight, gestational age, multiple pregnancy, mode of delivery, size for gestational age and site

72 hr deaths by subgroup	Intervention	Control	RR (95% CI) ¥
By birth weight category -- no./total no. (%)			
1.0 to <1.2kg	21/164 (12.8)	25/180 (13.9)	0.93 (0.55;1.57)
1.2 to <1.5kg	30/539 (5.6)	39/521 (7.5)	0.74 (0.47;1.17)
1.5 to <1.8kg	23/903 (2.5)	28/898 (3.1)	0.82 (0.48;1.42)
By gestational age category * -- no./total no. (%)			
<31 weeks	31/355 (8.7)	35/334 (10.5)	0.88 (0.55;1.41)
31 to <34 weeks	12/644 (1.9)	26/696 (3.7)	0.48 (0.25;0.94)
34 to <37 weeks	8/441 (1.8)	17/421 (4.0)	0.45 (0.2;1.04)
>=37 weeks	4/139 (2.9)	3/130 (2.3)	1.37 (0.32;5.89)
By mode of delivery -- no./total no. (%)			
Vaginal birth	47/1047 (4.5)	57/986 (5.8)	0.77 (0.53;1.13)
C-section	27/559 (4.8)	35/613 (5.7)	0.87 (0.53;1.43)
By single/twin infants-- no./total no. (%)			
Singleton	59/1176 (5.0)	70/1170 (6.0)	0.84 (0.6;1.17)
Twins	15/430 (3.5)	22/429 (5.1)	0.70 (0.36;1.36)
By size for gestational age* -- no./total no. (%)			
Small for GA	18/637 (2.8)	23/618 (3.7)	0.76 (0.41;1.39)
Not small for GA	36/941 (3.8)	58/963 (6.0)	0.64 (0.42;0.96)
By site-- no./total no. (%)			
Ghana	17/205 (8.3)	15/205 (7.3)	1.13 (0.58;2.22)
India	22/693 (3.2)	27/682 (4.0)	0.80 (0.46;1.39)
Malawi	21/217 (9.7)	26/220 (11.8)	0.82 (0.47;1.44)
Nigeria	2/108 (1.9)	7/107 (6.5)	0.28 (0.06;1.34)
Tanzania	12/383 (3.1)	17/385 (4.4)	0.71 (0.34;1.46)

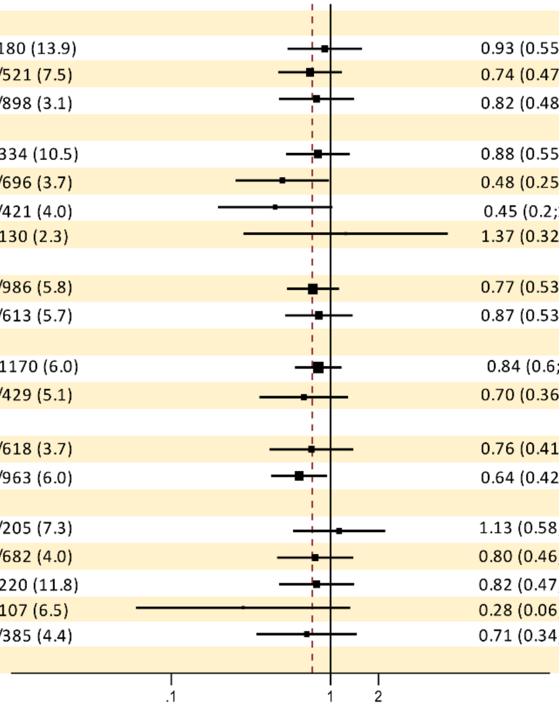

** 27 infants in the intervention and 18 infants in the control group have their gestational age at birth missing. Size for gestational age could not be calculated for one additional infant that was born with indeterminate sex.*

¥ adjusted by site and the clustering due to multiple births. For the subgroup analysis by site, adjustment was only done for the clustering effects of infants born to same mother in case of twins.

***The widths of the confidence intervals were not adjusted for multiplicity, so the intervals should not be used to infer definitive intervention effects. The size of the squares representing the point estimates is proportional to the weight assigned to the group.*

Table S1. Primary and secondary outcomes

Primary outcomes	Description	Measurement strategy
A. Death between enrolment and 72 hours of age	Death of an enrolled neonate occurring any time between enrolment and 72 hours of age.	Vital status recorded every 12 hours during hospital stay (enrolled baby unlikely to be discharged before 72 hours of age)
B. Death between enrolment and 28 days of age	Death of an enrolled neonate occurring any time between enrolment and 28 days of age.	Vital status recorded every 12 hours during hospital stay, and at a home visit on day 29 of age.
**Secondary outcomes**	**Description**	
Exclusive breastfeeding (or exclusive breast milk feeding) at the end of the neonatal period.	Exclusive breastfeeding is defined as an infant receiving only breast milk and no other liquid or solid, with the exception of vitamin or mineral supplements, medicines or ORS, if prescribed.	24 hour feeding recall at a home visit on day 29 of age.
Fully breastfed at discharge	The baby that could feed fully by suckling on the breast, without requiring any feeding by cup or nasogastric tube at the time of discharge.	Mode of feeding recorded every 12 hours during hospital stay
Suspected sepsis	Sepsis will be suspected when a baby ha clinical deterioration after initial improvement. This includes increase in respiratory distress after initial improvement, lethargy after improvement in activity, fever, or hypothermia after baby started maintaining temperature (not associate with environment hypothermia or with hypoglycemia).	Clinical signs recorded every 12 hours during hospital stay
Hypothermia	Any instance of axillary temperature <36°C from 2h after randomization until discharge (or 28 days of age if not discharged until then).	Temperature recorded every 12 hours during hospital stay

Hypoglycemia	Any measure of blood glucose <45 mg/dl (2.6mmol/l).	At mandatory measures at 6, 12, 18 and 24 hours of age, or at any other time if needed upto 36 hours of age
Time to clinical stabilization	Age at which the baby is considered to be clinically stable. The following criteria for stability should be met for at least a continuous period of 24 hours:<br>(i) Respiratory rate 40-60/min<br>(ii) No apnoea<br>(iii) No need for CPAP<br>(iv) SpO2 on room air >90%<br>(v) Heart rate 80-18 0/min<br>(vi) Axillary temperature 36-37.4°C<br>(vii) No need for IV fluids	Clinical signs and health care recorded every 12 hours during hospital stay
Death in babies not enrolled in the study up to 72 hours of age	Death between birth and 72 hours of age of a neonate born in the hospital who had a birth weight between 1.0 to<1.8kg but could not be enrolled in the study.	Hospital mortality data for all babies with birth weight between 1.0 to<1.8kg who died before screening, or had an exclusion criterion.
Maternal satisfaction with health care in the hospital	Satisfaction with health care received by the mother and baby.	Questionnaire to the mother after transfer to KMC ward, and around the time of discharge from the hospital.
Maternal depression	Patient Health Questionnaire 9 (PHQ-9) score of 15 points or more.	PHQ-9 administered to mothers at the day 29 home visit (Kroenke 2011).

Table S2. Additional baseline characteristics of randomized infants, mothers and households

	Immediate Kangaroo Mother Care	Control
**Infant's characteristics#**	**N=1609**	**N=1602**
Gestational age at birth category, n (%)		
<34 weeks	1000 (63.2)	1032 (65.1)
34 to <37 weeks	443 (28.0)	421(26.6)
≥37 weeks	139 (8.8)	131 (8.3)
Birth weight category, n (%)		
1.0 to <1.5 kg	704 (43.8)	703 (43.9)
1.5 to <1.8 kg	905 (56.2)	899 (56.1)
Apgar score at 5 min after birth, n (%) ¥		
<4	10 (0.6)	6 (0.4)
≥4 to <7	120 (7.5)	129 (8.1)
≥7	1479 (91.9)	1465 (91.5)
**Mother's clinical characteristics#**		
Number of mothers	1470	1474
Last available haemoglobin g/dl, mean (SD)¶	10.7 (1.7)	10.8 (1.7)
Eclampsia at time of birth, n (%)	7 (0.5)	9 (0.6)
Rupture of membranes for >18h before birth, n (%) ¶	212 (14.9)	203 (14.3)
Foul smelling liquor, n (%) ¶	24 (1.7)	16 (1.1)
Fever (>38°C) during labour or childbirth, n (%) ¶	5 (0.3)	6 (0.4)
**Socio-economic household characteristics**		
Number of households	1470	1474

Mother never been to school, n (%) §	155 (10.6)	138 (9.4)
Father never been to school, n (%) §	94 (6.8)	81 (5.8)
Father's median years of schooling, median (IQR)§	10 (8,12)	11 (8,12.5)

# There were 534 infants (from 267 mothers) who were born from a multiple pregnancy and both were eligible and enrolled (278 infants in the intervention and 256 infants in the control).
In addition, there were 325 mothers with multiple pregnancies in whom only one of the infants was eligible and the other one was ineligible (152 infants in the intervention group and 173 in the control).

** Gestational age based on ultrasound in first or second trimester, and if not available then based on LMP, and if both USG and LMP not available, then based on Ballard score(assessing measures of maturity on examination)*[21]

¥ Gestational age at birth missing for 27 infants in intervention and 18 infants in control group Apgar score 5 min after birth missing for 2 infants in the control group

¶ Haemoglobin level missing for 322 mothers in intervention and 342 in control group
Rupture of membranes >18hs before birth information missing for 47 mothers in intervention and 51 in control group.
Foul smelling liquor information missing in 19 mothers in intervention and 22 mothers in control group.
Fever during labour or childbirth information missing for 6 mothers in intervention and 14 in control group

§2 households in intervention and 2 in control group have missing data on mother's education
80 households in intervention and 87 in control group have missing data on father's education

Table S3. Duration of skin-to-skin contact in hours per day while the newborns were in the NICU during the first 2 weeks of life

	**Immediate Kangaroo Mother Care**		**Control**	
	N	Median (IQR)	N	Median (IQR)
Day 1	1609	18.7 (13.4;20.5)	1602	0 (0;0)
Day 2	1558	20.1 (14.7;21.5)	1534	0 (0;2.0)
Day 3	1441	18.3 (9.5;20.9)	1377	0.5 (0;2.9)
Day 4	1277	16.1 (7.2;20.5)	1187	0.8 (0;3.0)
Day 5	1110	16.5 (8.4;20.5)	1016	1.2 (0;3.4)
Day 6	941	17.2 (8.7; 20.6)	865	1.4 (0;3.8)
Day 7	769	18.3 (9.3;20.8)	739	1.7 (0;5.6)
Day 8	615	19.1 (11.5;21.0)	620	1.8 (0; 6.0)
Day 9	475	19.7 (12.5;21.1)	536	1.9 (0; 6.8)
Day 10	382	19.7 (12.5;21.3)	445	1.9 (0;7.4)
Day 11	317	19.9 (13.2;21.3)	366	2.0 (0;7.0)
Day 12	276	19.9 (14.5;21.5)	311	1.9 (0;6.5)
Day 13	244	20.0 (15.3;21.3)	267	2.0 (0;7.3)
Day 14	201	20.4 (15.5;21.3)	232	2.0 (0;8.3)

Table S4. Neonatal mortality in Intervention group by hours of skin-to-skin contact per day

**Hours of skin-to-skin contact per day in first 72 hours of life**	**Intervention group**	**Intervention group (excluding infants with signs of severe illness in first 6 hr of life) ***
Death between enrolment and 28 days of age, n (%)	N=1596	N=1466
<10 hr§	76/243 (31.3%)	42/190 (22.1%)
≥10 -19 hr	100/894 (11.2%)	91/835 (10.9%)
≥20 hr	15/459 (3.3%)	14/441 (3.2%)
Deaths between enrolment and 72 hr of age, n (%)	N=1606	N=1474
<10 hr §	47/244 (19.3%)	21/191 (11.0%)
≥10 -19 hr	27/897 (3.0%)	23/837 (2.7%)
≥20 hr	0/465 (0.0%)	0/446 (0.0%)

** Severe signs of illness in first 6 hr include any of the following: SpO_2<85%, grunting, no movement, CRT>3sec, Temperature<35.5ºC*

§ 16 infants never initiated skin-to-skin contact. Of those, 10 showed severe signs of sickness before 6 hr and were excluded from the subgroup analysis.

Table S5. Cause-specific neonatal mortality in randomized infants

Cause of death	Immediate Kangaroo Mother Care n= 1596	Control n= 1587	RR (95% CI)
Sepsis, n (%)	70 (4.4%)	109 (6.9%)	0.64 (0.48–0.86)
Preterm birth complications*, n %)	79 (4.9%)	83 (5.2%)	0.95 (0.70–1.28)
Perinatal asphyxia, n (%)	12 (0.8%)	18 (1.1%)	0.66 (0.32–1.37)
Congenital malformation, n (%)	10 (0.6%)	10 (0.6%)	0.99 (0.42–2.38)
Other specific cause, n (%)	4 (0.3%)	5 (0.3%)	0.80 (0.21–2.96)
Sudden death, n (%)	16 (1.0%)	20 (1.3%)	0.80 (0.41–1.53)
Undetermined, n (%)	0	4 (0.3%)	-

**Respiratory distress syndrome, intraventricular hemorrhage or necrotizing enterocolitis*

Table S6. Additional breastfeeding outcomes (post-hoc analyses)

Outcome	Immediate Kangaroo Mother Care (n=1609)	Control (n=1602)	RR (95% CI)
Initiation of breastmilk feeds within 24 hr, n (%)	941 (58.5%)	729 (45.5%)	1.29 (1.20–1.37)
Infant put to breast before 72 hr of age, n (%)	1108 (68.9%)	832 (51.9%)	1.32 (1.24–1.41)
Age Infant first put to the breast in hr, median (IQR)	41 (21–83)	66 (36–138)	1.50 (1.40–1.62)*
Reached full breastmilk feeds within 7d, n (%)	1261 (78.4%)	1105 (69.0%)	1.14 (1.09–1.19)
Discharge on exclusive breastmilk feeding**, n (%)	1208 (93.1%)	1067 (88.7%)	1.05 (1.02–1.08)

** Hazard ratio*

*** only among discharged infants (1298 intervention; 1203 control)*

Table S7. Risk factors for low birth weight in the pre-Screening form (any one of the following)

No.	Risk Factor
1	Estimated gestation of less than 37 weeks
2	Evidence of intra uterine growth restriction based on second-or-third trimester ultrasound
3	Fundal height of less than 32 cm
4	Age less than 18 years
5	Height of less than 1.5 m
6	Multiple pregnancy
7	Pre-eclampsia or eclampsia
8	Severe anemia, (< 7 g/dl)
